# Recommendations for the management of indeterminate HIV PCR results within South Africa’s early infant diagnosis programme

**DOI:** 10.4102/sajhivmed.v17i1.451

**Published:** 2016-05-13

**Authors:** Ahmad Haeri Mazanderani, Karl-Günter Technau, Nei-Yuan Hsiao, Jean Maritz, Sergio Carmona, Gayle G. Sherman

**Affiliations:** 1 Centre for HIV & STIs, National Institute for Communicable Diseases, South Africa; 2 Department of Medical Virology, University of Pretoria, South Africa; 3 Empilweni Services and Research Unit, Johannesburg, South Africa; 4 Department of Paediatrics and Child Health, University of the Witwatersrand, South Africa; 5 Division of Medical Virology, University of Cape Town, South Africa; 6 National Health Laboratory Service, South Africa; 7 Division of Medical Virology, Department of Pathology, Stellenbosch University, South Africa; 8 Department of Molecular Medicine and Haematology, University of the Witwatersrand, South Africa

## Abstract

Indeterminate HIV PCR results represent missed diagnostic opportunities within South Africa’s early infant diagnosis programme. These results not only delay diagnosis and appropriate management but are also a source of confusion and apprehension amongst clinicians and caregivers. We describe the extent of indeterminate HIV PCR results within South Africa’s early infant diagnosis programme and provide recommendations for the management of these cases, both in terms of laboratory practice and the clinical care of the infants.

## Introduction

Early infant diagnosis (EID) of HIV using highly sensitive polymerase chain reaction (PCR) methods and rapid linkage for the treatment of those who test positive is considered the gold standard of paediatric HIV care. Failure to initiate prompt combination antiretroviral therapy (cART) in an HIV-infected infant has been associated with considerable morbidity and mortality.^1,2^ The peak mortality rate for HIV-infected infants has been found to occur before 3 months of age in South Africa, emphasising the importance of rapid access to treatment.^3^ Hence, an early definitive test result indicating either a positive or negative HIV status is imperative. Indeterminate HIV PCR results, which can occur at all ages of testing (i.e. between birth and 18 months), represent missed diagnostic opportunities where the result is neither clearly positive nor negative. This not only delays diagnosis and appropriate management but is also a source of confusion and apprehension amongst clinicians and caregivers. We describe the extent of indeterminate HIV PCR results within South Africa’s EID programme and provide recommendations for the management of these cases, both in terms of laboratory practice and the clinical care of the infants. The purpose of these recommendations is to provide guidance for laboratory staff and the relevant clinical care providers on managing indeterminate HIV PCR results and to standardise practice within the National Health Laboratory Service (NHLS) EID laboratories.

## Early infant diagnostic testing within the South African public sector

Whereas previously the South African National Department of Health recommended routine HIV PCR testing at 6 weeks of age for HIV-exposed infants, new guidelines published on 01 June 2015 state that all HIV-exposed infants should have an HIV PCR test at birth, 10 weeks of age and 6 weeks after stopping breastfeeding if still under 18 months of age at that time.^4^ In children receiving prolonged nevirapine prophylaxis up to 12 weeks of age, an additional HIV PCR test is required at 18 weeks of age. The current guidelines recommend confirming the HIV status of all infants with a positive HIV PCR result by repeating the HIV PCR test on a second specimen.^4^

Two types of specimens can be used for HIV PCR testing. The most common specimen used is capillary blood from a heel prick spotted onto a cotton-based paper card, which is dried at the site of collection. This is known as a dried blood spot (DBS) and requires three full spots per card. Anti-coagulated ethylenediaminetetraacetic acid (EDTA) whole blood (purple top tube) with the minimum volume of 250 µL (0.25 mL) is also a suitable specimen.

Since 2010, all EID laboratories within the NHLS have used the same HIV PCR assay. The COBAS^®^ AmpliPrep/COBAS^®^ TaqMan (CAP/CTM) HIV-1 Qualitative Test (Roche Molecular Systems, Inc., Branchburg, NJ) is a total nucleic acid real-time reverse transcriptase PCR assay that detects HIV-1 proviral DNA and HIV-1 RNA on EDTA whole blood or DBS specimens.^5^ A new version of the assay, CAP/CTM v2.0, was introduced during the course of 2014. This version replaces the CAP/CTM test, is approved for *in vitro* diagnostic use and has improved analytical sensitivity.^6^ Whereas the previous version of the assay was found to have a limit of detection of 1090 RNA copies/mL using 60 µL DBS specimens, the CAP/CTM v2.0 has a reported limit of detection of 300 RNA copies/mL.^6,7^

## Indeterminate HIV PCR results

An indeterminate result means that the HIV PCR test yielded a valid but inconclusive result that is interpreted as being neither clearly positive nor negative. The term ‘equivocal’ was used in the past to qualify HIV PCR results of uncertain significance but is no longer used in NHLS EID laboratories. Indeterminate results have a detectable target, as determined by the instrument, but the amplified viral signal is of such a low level that it could potentially be a false-positive result. Standard operating procedures (SOPs) within the NHLS define results as ‘indeterminate’ according to specific real-time PCR parameters. The cut-off criteria are based on laboratory findings of poor positive predictive value and irreproducible positive results associated with higher cycle threshold (Ct) and lower relative fluorescence intensity (RFI) values.^8,9^ Currently, the NHLS’ national EID SOP defines an indeterminate HIV PCR result as a result with a detected target that has a Ct value > 33 and/or RFI < 5. These cut-off criteria will be continuously reviewed and, as they can potentially be influenced by a number of clinical, pre-analytic and analytic considerations, are likely to change with time. These variables include the type of specimen tested (i.e. DBS versus EDTA whole blood), reduction in mother-to-child transmission rate (i.e. reduced background prevalence) and the potential for antiretroviral prophylaxis to impact on diagnostic sensitivity.

## Extent of indeterminate results

There are approximately 270 000 HIV-exposed infants born each year in South Africa.^10,11^ Whereas this number is thought to be fairly constant, the volume of testing has increased year on year throughout the country. In 2014, 375 469 HIV PCR tests were performed, equating to an estimated testing coverage of 85% with approximately 1.8% of infants testing positive at 6 weeks of age (personal communication Prof Gayle Sherman).^12^ Indeterminate results are relatively rare and represent less than 1% of all registered specimens within the South African EID programme, on average amounting to less than 300 specimens per month. However, since 2012 indeterminate results have consistently comprised greater than 16% of all detected specimens (i.e. all positive and indeterminate specimens combined). Hence, indeterminate results represent a significant proportion of infants requiring urgent follow-up within the EID programme.

## Clinical and laboratory management of indeterminate results

The management of infants with indeterminate results is distinct from those with a positive result and requires a multidisciplinary approach from laboratories, pathologists, clinicians and programme managers. Depending on the referral structures in each district, the primary clinician should urgently seek advice for each case from more specialised clinicians, such as District Clinical Specialist Team paediatricians and paediatric infectious disease specialists. Furthermore, pathologists based at the NHLS EID laboratories should be consulted, and prevention of mother to child transmission (PMTCT) and HIV and/or AIDS, STIs and TB (HAST) programme managers should be contacted.

## Laboratory management

Indeterminate results, as defined by the NHLS’ EID SOP, should be treated as urgent and reviewed by an appropriately trained and experienced laboratory staff member, preferably a registrar or pathologist. The Ct and RFI values should be entered on the laboratory information system and the laboratory information system should be searched for previous HIV PCR and HIV viral load (VL) test results. Furthermore, the contact clinician who requested the HIV PCR test and/or a designated centralised responsible person for the district or province (e.g. District Clinical Specialist Team paediatrician or paediatric infectious disease specialist or PMTCT coordinator or HAST programme manager) should be contacted to discuss the case and requested to submit repeat samples where appropriate.

## Clinical management

Every primary clinician should have contact details of specialist clinicians, programme managers and their NHLS EID laboratory from the outset. Accurate completion of the NHLS requisition form, with patient and clinician contact details, facilitates this multidisciplinary approach and should include the data set listed in Box 1. Special care should be taken to ensure that the details on the request form reflect those on the specimen (i.e. ensure that the name, surname and barcode on the form and on the specimen are the same).

BOX 1 HIV PCR request form details.

**The following details of the infant being tested should be entered on the laboratory requests form and captured on the laboratory information system**:

- Clinic or hospital name
- Name and surname of patient
- Date of birth
- Gender
- File number
- Patient address and contact details
- Specimen type and collection date
- Healthcare workers name, registration number and contact details.

**In addition to the above, the following details when entered on the request form should be captured on the laboratory information system:**

- Infant’s RSA identity number
- Road to Health Booklet number.

**FIGURE 1:**
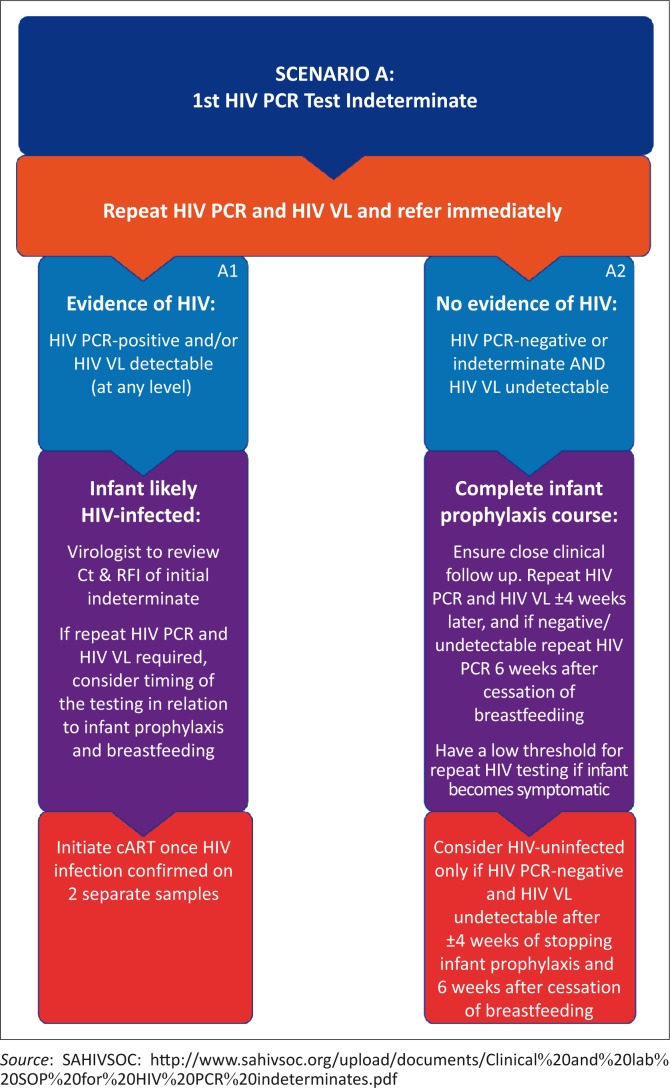
Scenario A.

**FIGURE 2:**
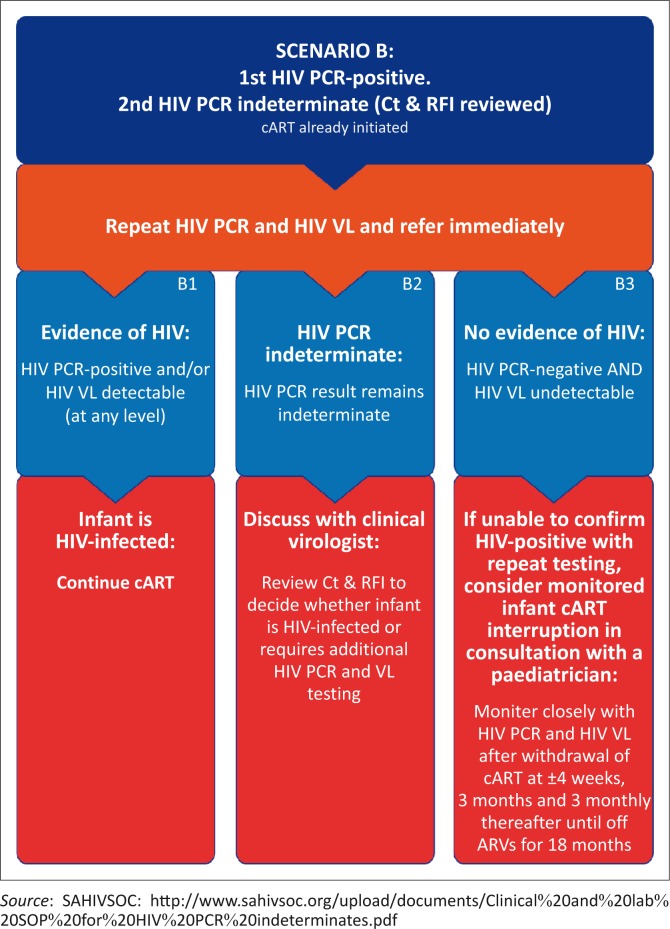
Scenario B.

The actions required following an indeterminate result are described in two broad scenarios. Scenario A outlines the management where an initial HIV PCR test, at any age between birth and 18 months, has an indeterminate result. Scenario B outlines the management where an initial HIV PCR test is positive, but the confirmatory HIV PCR is indeterminate (Figures 1 and 2, respectively).

### Scenario A

#### The first HIV PCR test has an indeterminate result

A specimen for repeat HIV PCR testing and an additional specimen for HIV VL testing should be submitted immediately and the patient referred.^4^ Referral can mean seeking advice from clinicians and/or pathologists or sending the patient to a specialist referral centre urgently. Importantly, appropriate referral should not be delayed whilst awaiting the laboratory results of the repeat HIV PCR and HIV VL tests.

Where the repeat HIV PCR test is positive and/or HIV VL is detectable (i.e. any value above the detection limit of the assay), the child is likely HIV-infected (Figure 1: A1). Infant cART initiation should not be delayed by further testing. Although these cases require a confirmatory HIV PCR and/or HIV VL to definitively establish a positive HIV infection status, the clinical team must consider each case individually. In some cases, an indeterminate HIV PCR result, depending on Ct/RFI values, followed by a positive HIV PCR and/or detectable HIV VL result may be sufficient to establish a diagnosis of HIV infection. If not, another specimen for confirmatory HIV PCR and HIV VL tests is required at the time of cART initiation.

Where the repeat HIV PCR is negative or indeterminate again and the HIV VL is undetectable, it is important to consider that HIV infection cannot be excluded in the presence of antiretroviral prophylaxis (e.g. daily nevirapine [NVP]) or within 4 weeks of discontinuing prophylaxis (Figure 1: A2). As antiretroviral therapy (ART) may suppress the target to less than detectable levels, it is important to complete the infant ART prophylaxis course and repeat HIV PCR and HIV VL 4 weeks later. The infant should be monitored clinically every 2 weeks prior to this, and if the child becomes symptomatic for HIV infection, testing should be repeated immediately. Healthcare workers should have a low threshold for repeat HIV PCR testing at any opportunity before 10–18 weeks of age.

### Scenario B

#### The first HIV PCR is positive but the second, confirmatory HIV PCR is indeterminate

A specimen for repeat HIV PCR testing and an additional specimen for HIV VL testing should be submitted immediately and the patient referred. Where the patient has already been initiated on cART, as per guidelines, this should not be interrupted. Appropriate referral should not be delayed whilst awaiting the laboratory results of the repeat HIV PCR and HIV VL tests.

Where the repeat HIV PCR is positive and/or HIV VL is detectable (i.e. any value above the detection limit of the assay), the child is confirmed HIV-infected on account of HIV having been detected twice on separate samples (Figure 2: B1). It is imperative that such patients continue receiving cART.

Where the repeat HIV PCR is indeterminate and HIV VL is undetectable, the Ct and RFI should be reviewed in consultation with a pathologist (e.g. clinical virologist) to decide whether the infant can be considered HIV-infected or whether HIV PCR and HIV VL require repeat testing (Figure 2: B2).

Where the repeat HIV PCR is negative and the HIV VL is undetectable, it is important to consider that HIV infection cannot be excluded in the presence of antiretroviral prophylaxis (daily NVP) or cART if already initiated (Figure 2: B3). The best approach for these infants should be determined within the multidisciplinary team. It is vital to keep the patient’s caregiver informed and supported (see ‘Counselling Suggestions’ below) and the patient kept in close clinical follow-up. The same approach should be followed for infants with repeatedly indeterminate HIV PCR results.

In all cases, a clear plan should be documented, communicated and adhered to. If the diagnosis remains unclear despite all attempts at resolution, the last resort is a monitored treatment interruption under the guidance of an experienced paediatric HIV clinician, if treatment has been started. It is recommended that follow-up testing be performed at 1 month, 3 months, and 3 monthly thereafter for a minimum of 18 months off ART.

## Counselling of caregivers

The mother or primary caregiver should be consulted regarding the further management and follow-up of an infant who has received an HIV PCR indeterminate result. The decision to initiate cART, when indicated, must consider the practical implications of where and how treatment will be continued. Infant feeding should be carefully discussed considering that breastfeeding improves outcome in HIV-infected infants, and maternal adherence to ART during breastfeeding should be stressed. All cases should urgently be brought to the attention of the relevant HIV clinic. Engagement of the family should be encouraged but the mother or primary caregiver should guide the level of family involvement. The mother’s well-being should be monitored by providing adequate ART care, TB screening and adequate psychosocial support. It is important to document discussions with the mother in the infant’s bed letter and road to health booklet. The mother should have the clinic contact numbers and clinical course and decisions should be documented in the infant’s road to health booklet to facilitate communication between different healthcare providers.

Where possible, to improve compliance, continuity of care should occur at a single facility, preferably with a single healthcare worker.

The guiding principles of counselling in these cases should include:

- The mother or primary caregiver must be involved with honest and frank information at every stage.
- The message must be communicated that there is a team involved with the infant’s care, that guidelines and resources exist to determine the final outcome. However, the length of this process is uncertain. Follow-up care and clear communication, both verbal and written, is critical especially for mobile mothers.
- The team may not know the answer to the diagnostic dilemma at present but is aware how stressful this is and will undertake to find the solution in consultation with the mother and the necessary experts. At this stage, it is critical that the follow-up care is monitored and tracked to reassure the mother or family that somebody is pursuing the problem. In the absence of a clear answer, this should provide some level of relief.
- A clear plan should be documented, communicated and adhered to. In the event of an unclear diagnosis despite all attempts to come to a clear solution, the last resort will be a monitored treatment interruption, if the infant is on cART. It is recommended that follow-up testing be performed at 1 month, 3 months and 3 monthly thereafter for a minimum of 18 months off ART.

Note that these families need increased adherence support as they may be confused by the indeterminate results and the lack of a final confirmed diagnosis may contribute to poor adherence to ART.

## Summary

The laboratory diagnosis of HIV in infants less than 18 months of age requires two HIV PCR-positive results, each on a separate specimen, as per South Africa’s National Consolidated Guidelines of 01 June 2015. Alternatively, one HIV PCR-positive result in association with an HIV VL that is detectable on a separate specimen is also diagnostic of HIV. An indeterminate HIV PCR result means that the test is inconclusive (i.e. it is not clearly positive or negative). Patients with indeterminate results require immediate further testing, to determine whether the infant is HIV-infected, and referral. Repeat HIV PCR and HIV VL testing needs to be performed as a matter of urgency and the patient managed according to the algorithm outlined in this recommendation (Figures 1 and 2). Infants in whom the diagnosis of HIV remains inconclusive or where discordant results have been obtained (i.e. a positive HIV PCR followed by a negative HIV PCR and undetectable HIV VL) need to be managed by a multidisciplinary team and should be discussed as a matter of urgency with a specialist clinician and pathologist. Repeat HIV testing and clinical monitoring is required until an HIV status is established. It is important to remember that infants cannot be considered HIV-uninfected unless repeat testing occurs at least 4 weeks after cessation of infant prophylaxis and 6 weeks after cessation of breastfeeding. Counselling the mother or primary caregiver regarding the indeterminate result is of paramount importance to ensure successful follow-up and arrival at a definitive diagnosis.

## References

[1] Newell ML, Coovadia H, Cortina-Borja M, et al Mortality of infected and uninfected infants born to HIV infected mothers in Africa: A pooled analysis. Lancet. 2004;364:1236–1243. http://dx.doi.org/10.1016/S0140-6736(04)17140-7 15464184 10.1016/S0140-6736(04)17140-7

[2] Violari A, Cotton MF, Gibb DM, et al Early antiretroviral therapy and mortality among HIV-infected infants. N Engl J Med. 2008;359:2233–2244. http://dx.doi.org/10.1056/NEJMoa0800971 19020325 10.1056/NEJMoa0800971 PMC2950021

[3] Bourne DE, Thompson M, Brody LL, et al Emergence of a peak in early infant mortality due to HIV/AIDS in South Africa. AIDS. 2009;23:101–106. http://dx.doi.org/10.1097/QAD.0b013e32831c54bd 19065753 10.1097/qad.0b013e32831c54bd

[4] National Department of Health National consolidated guidelines for the Prevention of Mother-to-Child Transmission of HIV (PMTCT) and the Management of HIV in Children, Adolescents and Adults. Pretoria: Department of Health; 2014.

[5] Roche^®^ COBAS^®^ AmpliPrep/COBAS^®^ TaqMan HIV-1 Qual Test [package insert]. Branchburg, NJ: Roche; 2007.

[6] Roche^®^ COBAS^®^ AmpliPrep/COBAS^®^ TaqMan HIV-1 Qualitative Test, version 2.0 [package insert]. Branchburg, NJ: Roche; 2013.

[7] Stevens W, Erasmus L, Moloi M, Taleng T, Sarang S Performance of a novel human immunodeficiency virus (HIV) type 1 total nucleic acid-based real-time PCR assay using whole blood and dried blood spots for diagnosis of HIV in infants. J Clin Microbiol. 2008;46(12):3941–3945. http://dx.doi.org/10.1128/JCM.00727-09 18923017 10.1128/JCM.00754-08 PMC2593254

[8] Maritz J, Preiser W, van Zyl GU Establishing diagnostic cut-off criteria for the COBAS AmpliPrep/COBAS TaqMan HIV-1 Qualitative test through validation against the Amplicor DNA test v1.5 for infant diagnosis using dried blood spots. J Clin Virol. 2012;53:106–109. http://dx.doi.org/10.1016/j.jcv.2011.12.002 22196872 10.1016/j.jcv.2011.12.002

[9] Maritz J, van Zyl GU, Preiser W Irreproducible positive results on the CobasAmpliprep/CobasTaqMan HIV-1 Qual test are different qualitatively from confirmed positive results. J Med Virol. 2014;86:82–87. http://dx.doi.org/10.1002/jmv.23811 10.1002/jmv.23811 24136657

[10] National Department of Health The 2011 National Antenatal Sentinel HIV & Syphilis Prevalence Survey in South Africa. Pretoria: Department of Health; 2012.

[11] Statistics South Africa Recorded live births 2011. Pretoria: Stats SA; 2012.

[12] Sherman GG, Lilian RR, Bhardwaj S, Candy S, Barron P Laboratory information system data demonstrate successful implementation of the prevention of mother-to-child transmission programme in South Africa. S Afr Med J. 2014;104(3 Suppl 1):235–238. http://dx.doi.org/10.7196/samj.7598 24893499 10.7196/samj.7598

